# Microbiota-associated antioxidant responses to selenium source and probiotic supplementation in beef cattle

**DOI:** 10.3389/fvets.2026.1882289

**Published:** 2026-07-20

**Authors:** Liang Xie, Wenhua Tan, Hui Li, Xia Wang, Jin Du, Xuerong Quan, Bingxin Guo, Rejun Fang

**Affiliations:** 1 College of Modern Agriculture, XiangXi Vocational and Technical College for Nationalities, Jishou, China; 2 College of Animal Science and Technology, Hunan Agricultural University, Changsha, China

**Keywords:** antioxidant status, beef cattle, growth performance, micro-ecological feed additives, rumen microbiota, selenium, serum biochemistry

## Abstract

Selenium (Se) and micro-ecological preparations are widely recognized for their roles in enhancing animal health, antioxidant capacity, and production performance. However, their combined effects and underlying mechanisms, particularly involving rumen microbiota, remain poorly understood. This study aimed to investigate the effects of different selenium sources (inorganic vs. organic) combined with micro-ecological probiotic supplementation on growth performance, serum biochemical parameters, antioxidant status, and rumen microbial composition in beef cattle. A total of 24 crossbred Xinjiang Brown steers (average initial body weight: 407.48 ± 36.10 kg; age: 19 months) were assigned to a 2 × 2 factorial design following a 14-day adaptation period and a 37-day feeding trial, with selenium source (sodium selenite vs. yeast selenium) and micro-ecological probiotic supplementation (0 vs. 3%). Growth performance, serum biochemical indices, antioxidant parameters, and rumen microbiota (16S rRNA sequencing) were analyzed. Weighted gene co-expression network analysis (WGCNA) was further employed to explore the associations between microbial modules and host phenotypes. Results showed that selenium source significantly affected feed conversion ratio (FCR), with sodium selenite resulting in improved feed efficiency compared to yeast selenium (*p* = 0.027). Selenium source significantly affected total antioxidant capacity (T-AOC; *p* = 0.043), malondialdehyde (MDA; *p* = 0.005), and glutathione peroxidase activity (GSH; *p* < 0.001), whereas probiotic supplementation significantly affected superoxide dismutase activity (SOD; *p* = 0.032) and GSH activity (*p* < 0.001). A significant interaction between selenium source and probiotic supplementation was observed for GSH activity (*p* = 0.002). WGCNA identified key microbial modules associated with T-AOC and GSH, within which genera such as Romboutsia, Paeniclostridium, and Turicibacter were significantly correlated with antioxidant indices. Further analysis suggested that selenium source may modulate growth performance through a microbiota–antioxidant axis, particularly via Romboutsia-mediated regulation of oxidative status. In conclusion, selenium source and micro-ecological probiotic supplementation jointly influence antioxidant capacity and rumen microbial structure, and may regulate feed efficiency through microbiota-mediated mechanisms. These findings provide new insights into nutritional strategies for improving beef cattle production.

## 1 Introduction

Trace elements, characterized as minerals accounting for less than 0.01% of animal body weight, include selenium (Se), copper (Cu), iron (Fe), manganese (Mn), zinc (Zn), and iodine (I). These elements fulfill indispensable roles in growth, production, reproduction, and sustenance of animal health and immune homeostasis (1, 2). Among them, selenium is exceptionally crucial due to its role in synthesizing various antioxidant enzymes and selenoproteins, such as glutathione peroxidase (GSH), thioredoxin reductase (TrxR), and selenoprotein P (SelP). In both animals and plants, selenium plays a vital part in regulating antioxidant defense, immune regulation, nutrient transport, and hormone synthesis (3–5). Appropriate selenium supplementation can markedly improve animal health and productive performance (6). Organic trace minerals encompass complexes or chelates generated through covalent or ionic bonds between metal elements and ligands such as proteins, small peptides, amino acids, organic acids, or polysaccharide derivatives. In animal production, they are commonly understood to denote additives that go beyond first-generation inorganic trace minerals. From a mechanistic perspective, organic trace minerals are transported in a chelated or complexed state via their ligands, thereby circumventing competitive antagonistic effects among metal ions during intestinal absorption. This enhances intestinal absorption efficiency, allowing lower inclusion rates to achieve higher efficacy and thus yielding higher bioavailability compared to inorganic forms (7). The superior efficacy of organic and bio-nanostructured selenium forms has been substantiated by recent research in dairy sheep. Conducted a 50 day feeding trial with lactating Barki ewes supplemented with sodium selenite or bio-nanostructured selenium (SeNSM) at 1.2 mg/kg dry matter. Compared with inorganic selenium, SeNSM tended to improve fiber digestibility, significantly enhanced blood glutathione peroxidase activity, and increased milk yield and milk component yields, as well as milk selenium content. Lambs from SeNSM-fed ewes also showed greater weight gains (8). The research supports further investigation of its interaction with other dietary modulators such as probiotics.

Micro-ecological preparations, alternatively termed micro-ecological preparations, are developed via the selection of beneficial and harmless micro-ecological probiotic strains or growth-promoting substances from natural environments in accordance with microbiological principles. These probiotics are cultivated and expanded using specialized techniques to produce live microbial products with high concentrations of viable bacteria capable of exerting beneficial effects on the host (9). Numerous studies have demonstrated that, in livestock and poultry production, micro-ecological preparations can boost growth performance, sustain intestinal microbial equilibrium, elevate meat quality, and augment disease resistance capabilities (10–12).

Different selenium sources (inorganic vs. organic selenium) may interact with micro-ecological preparations in both synergistic and antagonistic manners (13). Micro-ecological probiotics can enhance selenium bioavailability by improving intestinal absorption and facilitating the conversion of inorganic selenium into more bioactive organic forms (14). Conversely, selenium can modulate microbial community composition, selectively promoting beneficial microbes while suppressing pathogenic bacteria. Previous studies have indicated that selenium supplementation can alter rumen fermentation patterns and microbial structure, while micro-ecological probiotics such as Lactobacillus and *Saccharomyces cerevisiae* can improve rumen stability, enhance fiber digestion, and promote antioxidant status (15). However, excessive or unbalanced supplementation may also lead to microbial competition or metabolic redundancy, potentially limiting their combined benefits (16). Therefore, the interaction between selenium sources and micro-ecological probiotic supplementation is complex and remains insufficiently understood, particularly in terms of microbiota-mediated mechanisms.

In summary, both the trace element selenium and micro-ecological preparations have been validated to enhance antioxidant capacity, strengthen immune competence, and promote meat quality in animals. However, research on the combined use of selenium and micro-ecological preparations as dietary supplements in beef cattle production is still relatively understudied. Therefore, the present study was conducted to examine the effects of dietary supplementation with two different forms of selenium in combination with micro-ecological preparations on growth performance, serum biochemical parameters, and antioxidant indices in beef cattle, in order to offer a reference for the development of related products and their application in beef cattle production.

## 2 Materials and methods

### 2.1 Experimental materials

#### 2.1.1 Trace element selenium

Selenium sources were procured from Changsha Xingjia Bio-Engineering Co., Ltd. Sodium selenite had a selenium content of 1%, whereas yeast selenium had a selenium content of 0.2%.

#### 2.1.2 Micro-ecological preparation

The micro-ecological preparation was purchased from Changsha Xingjia Bio-Engineering Co., Ltd. containing ≥2 × 108 CFU/kg Lactobacillus and ≥3 × 107 CFU/kg *S. cerevisiae*, and additional auxiliary components.

### 2.2 Experimental materials

The experiment comprised a 14-day adaptation period, which was followed by a 37-day formal trial. A 2 × 2 factorial randomized design was adopted. Twenty-four healthy crossbred Xinjiang Brown steers with comparable body condition and an average initial body weight (IBW) of 407.48 ± 36.10 kg (age: 19 months) were randomly allocated to four treatment groups, with six replicates per group, and one animal per replicate.

In the concentrate supplement, all selenium-supplemented treatments were formulated to provide an equivalent dietary selenium level of 0.68 mg Se/kg complete diet. The selenium supplementation level was determined in accordance with the Chinese Feeding Standard for Beef Cattle (17) and was consistent with the dietary selenium level reported by Zhou et al. (18). Sodium selenite and yeast selenium, containing 1 and 0.2% elemental Se, respectively, were used as selenium sources. Accordingly, sodium selenite and yeast selenium were included at 68 mg/kg and 340 mg/kg complete diet, respectively. Treatment I received sodium selenite; Treatment II received sodium selenite together with 3% micro-ecological preparation; Treatment III received yeast selenium; and Treatment IV received yeast selenium together with 3% micro-ecological preparation. The micro-ecological preparation was supplemented at 3% of the concentrate mixture, corresponding to 1.09% of the complete diet on a dry matter basis, and contained ≥2 × 10^8^ CFU/kg Lactobacillus and ≥3 × 10^7^ CFU/kg *S. cerevisiae*. The probiotic supplementation level was selected according to the Chinese Feeding Standard for Beef Cattle National Feeding Standard for Beef Cattle. Diets for all groups were formulated in accordance with the National Feeding Standard for Beef Cattle (17), with a concentrate-to-roughage ratio of 37:63. The ingredient composition and nutritional levels of the diets are detailed in Table 1, and the nutrient levels of the roughage used in the trial are shown in Table 2.

**Table 1:** Ingredient composition and nutrient levels of the experimental diets (dry matter basis).

Item	Treatment I	Treatment II	Treatment III	Treatment IV
Ingredient composition (% of dry matter)				
Rice straw	19.04	19.04	19.04	19.04
Napier grass	9.35	9.35	9.35	9.35
Brewer’s grains	35.12	35.12	35.12	35.12
Corn	23.00	23.00	23.00	23.00
DDGS	3.65	3.65	3.65	3.65
Soybean meal	6.57	6.57	6.57	6.57
Palm oil	0.36	0.36	0.36	0.36
Wheat bran	1.09	0.00	1.09	0.00
Probiotic preparation	0.00	1.09	0.00	1.09
Premix^1^	1.82	1.82	1.82	1.82
Total	100.00	100.00	100.00	100.00
Nutrient levels^2^				
Crude protein/%	20.48	20.23	20.29	20.55
Neutral detergent fiber/%	47.32	46.59	47.65	46.71
Metabolizable energy/(MJ/kg)	9.19	9.38	9.1	9.39

^1^The premix provided per kilogram of complete feed: vitamin A, 14,000 IU; vitamin D, 1830 IU; vitamin E, 44840 IU; iron, 235.8 mg; copper, 25.4 mg; manganese, 80.3 mg; zinc, 140.9 mg; selenium, 0.68 mg; iodine, 1.2 mg; cobalt, 0.50 mg. ^2^Metabolizable energy values were calculated according to the Feeding Standard for Beef Cattle (NY/8152004), while all other values were determined by chemical analysis. This applies to all tables below.

**Table 2:** Nutrient composition of roughages (dry matter basis).

Item	Rice straw	Napier grass	Brewer’s grains
Crude protein/%	5.96	12.12	33.95
Neutral detergent fiber/%	73.31	62.01	62.69
Dry matter/%	90.77	14.86	27.91
Metabolizable energy/(MJ/kg)	3.32	4.26	5.73

### 2.3 Feeding management

Prior to the initiation of the experiment, the cattle barn was thoroughly disinfected. The steers were housed individually in pens and maintained under a stall-feeding system. All experimental steers were subjected to uniform management practices and fed at 09:00 and 16:00 daily, adhering to the feeding sequence whereby the concentrate mix was offered first, followed by the roughage. The quantity of concentrate mix supplied was equivalent to 1% of each animal’s live body weight. During the entire experimental period, animals had ad libitum access to both feed and fresh water. The pens were maintained in a clean and hygienic condition, with disinfection procedures conducted once weekly.

### 2.4 Determination indices and methods

#### 2.4.1 Growth performance

At the onset and conclusion of the experiment, steers were fasted for 12 h prior to body weight was measured to determine IBW and final body weight (FBW). The average daily gain (ADG) was calculated using IBW and FBW. During the last 5 days of the formal trial, the daily amount of feed offered and feed refusals were recorded for each animal to calculate the dry matter intake (TDMI), average daily feed intake (ADFI), and Feed conversion ratio (FCR) for each treatment group. The calculation formulas were as follows:

1. TDMI(kg) = Average daily feed intake ×Dry matter content of diet.
2. ADFI (kg/d) = TDMI / Number of experimental days.
3. ADG (kg/d) = (FBW − IBW)/ Number of experimental days.
4. FCR = ADFI / ADG.

#### 2.4.2 Determination of serum biochemical parameters

At the end of the experiment, three healthy steers with comparable body weight were randomly selected from each treatment group for blood sampling. Approximately 15 mL of blood was collected from the coccygeal vein with sterile vacuum blood collection tubes. Samples were centrifuged at 3,000 rpm for 10 min, and the serum was separated and stored at −80 °C for subsequent analysis. Serum concentrations of alanine aminotransferase (ALT), albumin (ALB), triglycerides (TG), total cholesterol (TC), total protein (TP), aspartate aminotransferase (AST), alkaline phosphatase (ALP), glucose (GLU), lactate dehydrogenase (LDH), and blood urea nitrogen (BUN) were measured using a ZY 450 automatic biochemical analyzer (Shanghai Kehua Bio-Engineering Co., Ltd.).

#### 2.4.3 Determination of serum antioxidant parameters

Serum total antioxidant capacity (T-AOC), GSH, superoxide dismutase (SOD) activity, catalase (CAT) activity, and malondialdehyde (MDA) concentration were determined using commercial assay kits (Nanjing Jiancheng Bioengineering Institute, Nanjing, China) in strict accordance with the manufacturer’s protocols.

#### 2.4.4 Rumen microbiota analysis

At the end of the feeding trial, rumen fluid samples were collected from the same 12 steers selected for serum biochemical and antioxidant analyses. Samples were collected before the morning feeding using a sterile oral stomach tube. To minimize saliva contamination, the initial portion of rumen fluid was discarded, and approximately 50 mL of rumen fluid was collected from each animal. The samples were filtered through four layers of sterile gauze, immediately placed into sterile centrifuge tubes, rapidly frozen in liquid nitrogen, and stored at −80 °C until microbial DNA extraction. Twelve healthy steers exhibiting comparable serum biochemical profiles and body condition were selected for rumen fluid collection and subsequent rumen microbiota analysis. Rumen microbial DNA was extracted and subjected to 16S rRNA gene sequencing targeting the V3–V4 region using the Illumina MiSeq/NovaSeq platform. Raw reads were processed using a standardized bioinformatics pipeline. Briefly, sequences were quality-filtered, denoised, and merged using DADA2 (19) to generate amplicon sequence variants (ASVs). Chimeric sequences were removed, and taxonomy was assigned using the SILVA database (20). The ASV table was aggregated at the genus level. Low-abundance taxa (present in <20% of samples or with relative abundance <0.01%) were filtered to reduce sparsity. Data were transformed into relative abundance and subsequently subjected to centered log-ratio (CLR) transformation after adding a pseudocount.

### 2.5 Data analysis

To ensure the consistency of subsequent serum biochemical, antioxidant, microbiota, and association analyses, 12 healthy steers exhibiting comparable serum biochemical profiles and body condition were selected for further data analyses. Correlation analysis among phenotypic traits was performed using the R package “corrplot,” and heatmaps were generated using “pheatmap.” Generalized linear models (GLM) were implemented using the “glm” function in R to evaluate the effects of serum parameters on growth performance. Principal component analysis (PCA) was conducted using the “prcomp” function, and visualization was performed using “ggplot2.”

A two-way analysis of variance (ANOVA) based on a univariate GLM was performed to assess the main effects of selenium source and micro-ecological probiotic supplementation, as well as their interaction, on growth performance, serum biochemical parameters, and microbial abundance. All results are expressed as mean ± standard deviation. Statistical significance was defined as *p* < 0.05, highly significant at *p* < 0.01.

For microbiome data, weighted gene co-expression network analysis (WGCNA) was conducted using the R package “WGCNA.” A soft-thresholding power was selected based on the scale-free topology criterion. Microbial modules were identified using hierarchical clustering, and module eigengenes (MEs) were correlated with serum biochemical and antioxidant parameters.

## 3 Results

### 3.1 Correlation patterns among growth performance, serum biochemical indices, and antioxidant parameters

**Figure 1:**
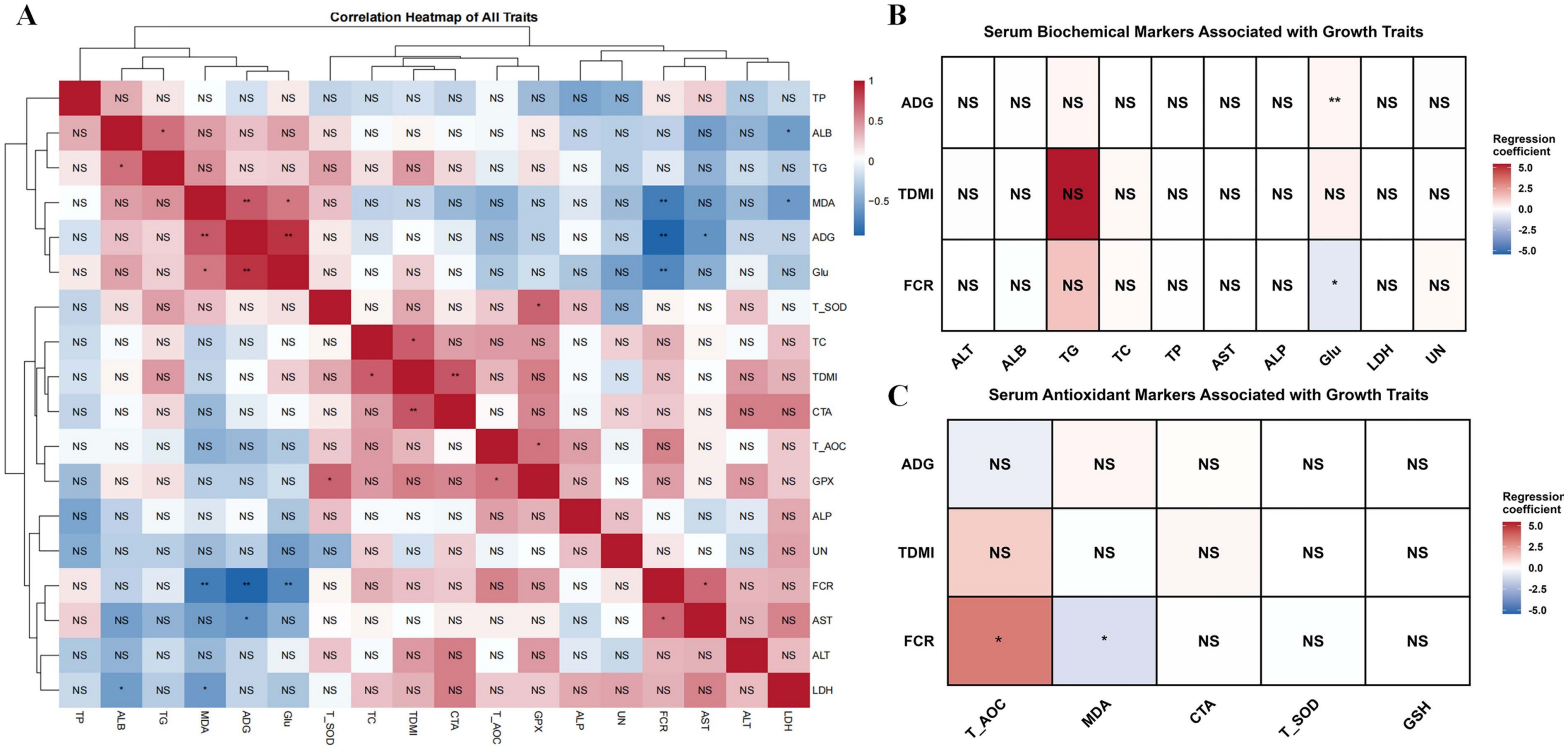
Relationships among phenotypic traits. **(A)** Correlation heatmap among growth performance, serum biochemical indices, and serum antioxidant parameters. NS indicates non-significant correlation; *indicates significant correlation; **indicates highly significant correlation. **(B)** Heatmap of regression coefficients of serum biochemical parameters on the three growth performance traits. NS indicates a non-significant regression coefficient; *indicates a significant regression coefficient. **(C)** Heatmap of regression coefficients of serum antioxidant parameters on the three growth performance traits. NS indicates a non-significant regression coefficient; *indicates a significant regression coefficient.

Different selenium sources and micro-ecological probiotic supplementation may influence growth performance by modulating metabolic processes, serum biochemical status, and antioxidant capacity. Therefore, correlation analysis was first performed among the three major growth performance traits, including ADG, TDMI, and feed conversion ratio (FCR), together with serum biochemical and antioxidant parameters. As shown in Figure 1A, the investigated phenotypic traits formed two major correlation clusters. One cluster mainly consisted of serum biochemical indices, whereas the other primarily included serum antioxidant parameters, suggesting that these two categories of traits may represent distinct but interconnected physiological processes. Among the serum-related indices associated with growth performance, ADG was significantly correlated with serum MDA and GLU concentrations. TDMI was associated with TC and T-AOC. FCR was significantly correlated with MDA, GLU, and AST. When focusing specifically on the three growth performance traits, ADG showed a highly significant correlation with FCR, whereas TDMI was not significantly associated with either ADG or FCR.

To further evaluate whether serum biochemical and antioxidant parameters could serve as potential explanatory factors for growth performance, regression analyses were performed. As shown in Figures 1B,C, serum GLU concentration had a positive effect on ADG and a negative effect on FCR, indicating that higher serum glucose availability may be associated with improved growth and feed efficiency. Among antioxidant parameters, T-AOC and MDA significantly affected FCR. Specifically, serum T-AOC was positively associated with FCR, whereas MDA was negatively associated with FCR. These results suggest that serum energy metabolism and oxidative status may be closely involved in regulating feed efficiency in beef cattle.

### 3.2 Effects of selenium source and micro-ecological probiotic supplementation on phenotypic traits

**Figure 2:**
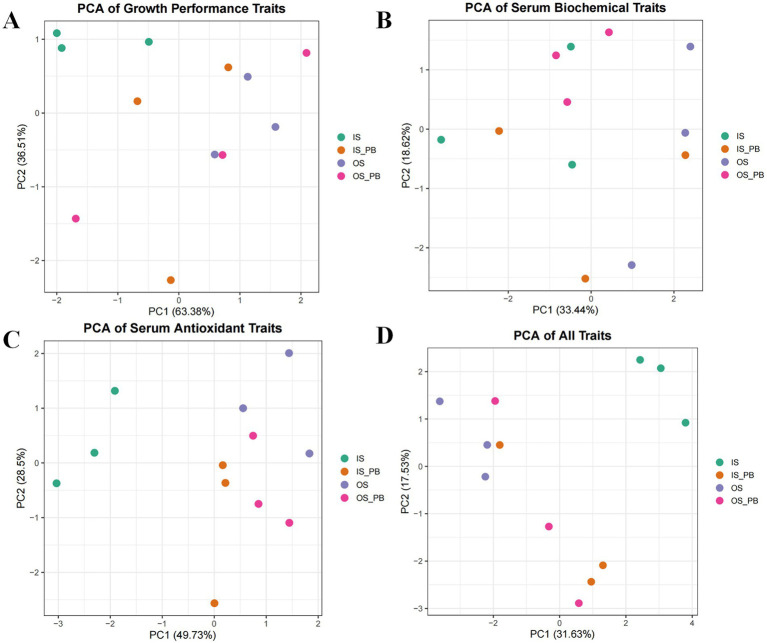
PCA analysis of phenotypic traits among different treatment groups. Each point represents one animal. IS, sodium selenite supplementation alone; IS_PB, sodium selenite combined with 3% micro-ecological probiotic preparation; OS, yeast selenium supplementation alone; OS_PB, yeast selenium combined with 3% micro-ecological probiotic preparation. **(A–D)** represent PCA plots based on growth performance traits, serum biochemical indices, serum antioxidant parameters, and all phenotypic traits, respectively.

To determine whether different selenium sources and micro-ecological probiotic supplementation affected phenotypic variation, PCA was performed separately for growth performance traits, serum biochemical indices, serum antioxidant parameters, and all measured phenotypes. The four treatment groups were defined as follows: IS, sodium selenite supplementation alone; IS_PB, sodium selenite combined with 3% micro-ecological probiotic preparation; OS, yeast selenium supplementation alone; and OS_PB, yeast selenium combined with 3% micro-ecological probiotic preparation. As shown in Figure 2A, PCA based on the three growth performance traits revealed that the IS group was separated from the other treatment groups, suggesting that sodium selenite alone had a distinct influence on growth-related phenotypes. In contrast, PCA based on serum biochemical indices showed no obvious separation among the four groups, indicating that selenium source and micro-ecological probiotic supplementation had relatively limited effects on overall serum biochemical profiles (Figure 2B). However, when serum antioxidant parameters were analyzed, samples from different treatment groups tended to cluster separately, suggesting that antioxidant traits were more sensitive to dietary treatments than serum biochemical indices (Figure 2C). When all phenotypic traits were considered together, individuals from the IS and OS groups tended to cluster within their respective groups, whereas samples from the two micro-ecological probiotic-supplemented groups showed partial overlap (Figure 2D). These results suggest that selenium source and micro-ecological probiotic supplementation jointly influenced host phenotypes, with the effect of sodium selenite alone being particularly evident.

To further quantify the effects of treatment factors, a two-way ANOVA based on the 2 × 2 factorial design was performed to evaluate the main effects of selenium source and micro-ecological probiotic supplementation, as well as their interaction, on individual traits and on the first principal component (PC1) of each trait category (Table 3). For growth performance traits, selenium source had no significant effect on ADG (*p* = 0.171) or TDMI (*p* = 0.291), but significantly affected FCR (*p* = 0.027). Compared with yeast selenium, sodium selenite resulted in a lower FCR. Probiotic supplementation did not significantly affect ADG (*p* = 0.996), TDMI (*p* = 0.127), or FCR (*p* = 0.370). The interaction between selenium source and probiotic supplementation was not significant for ADG (*p* = 0.301), TDMI (*p* = 0.229), or FCR (*p* = 0.084). Although ADG and TDMI did not differ significantly when analyzed separately, the sodium selenite groups showed numerically higher ADG and lower TDMI than the yeast selenium groups. Because FCR was calculated for each individual animal as TDMI divided by ADG before statistical analysis, the combined directional changes in TDMI and ADG resulted in a significant difference in FCR. These results indicate that selenium source was the major factor influencing feed efficiency in the present study. For serum biochemical indices, the overall effects of selenium source and micro-ecological probiotic supplementation were relatively limited. As shown in Table 4, micro-ecological probiotic supplementation significantly affected ALT (*p* = 0.013), whereas selenium source significantly affected LDH (*p* = 0.047). No significant effects were observed for most other biochemical indices or for the PC1 value of serum biochemical traits. This result was consistent with the PCA analysis, in which serum biochemical profiles did not show clear separation among treatment groups.

**Table 3:** Effects of selenium source and probiotic supplementation on growth performance of beef cattle.

Treatment group	Selenium source	Probiotic preparation (%)	ADG (kg/d)	TDMI (kg/d)	F/G	Growth_PC1
I	Sodium selenite	0	1.584 ± 0.103	7.950 ± 0.071	5.036 ± 0.376	−1.471 ± 0.848
II	Sodium selenite	3	1.482 ± 0.128	8.683 ± 0.685	5.869 ± 0.351	−0.001 ± 0.753
III	Yeast selenium	0	1.342 ± 0.067	8.544 ± 0.232	6.371 ± 0.241	1.099 ± 0.498
IV	YEAST selenium	3	1.445 ± 0.267	8.641 ± 0.431	6.080 ± 0.808	0.372 ± 1.913
Main effect	Sodium selenite		1.533 ± 0.118	8.316 ± 0.592	5.453 ± 0.560	−0.736 ± 1.078
	Yeast selenium		1.394 ± 0.183	8.593 ± 0.314	6.225 ± 0.557	0.736 ± 1.312
		0	1.463 ± 0.153	8.247 ± 0.360	5.704 ± 0.784	−0.186 ± 1.539
		3	1.464 ± 0.188	8.662 ± 0.512	5.974 ± 0.569	0.186 ± 1.316
*p*-value	Selenium source		0.171	0.291	0.027	0.056
	Probiotic preparation		0.996	0.127	0.37	0.588
	Selenium source + probiotic preparation		0.301	0.229	0.084	0.134

**Table 4:** Effects of selenium source and probiotic supplementation on serum biochemical indices of beef cattle.

Treatment group	Selenium source	Probiotic preparation/%	ALT (u/L)	ALB (g/L)	TG (mmol/L)	TC (mmol/L)	TP (g/L)	AST (u/L)	ALP (u/L)	GLU (mmol/L)	LDH (u/L)	BUN (mmol/L)	Serum_PC1
I	Sodium selenite	0	22.090 ± 0.820	37.683 ± 2.791	0.195 ± 0.054	4.656 ± 0.587	70.730 ± 3.466	93.730 ± 31.416	151.120 ± 27.641	4.459 ± 0.161	1228.533 ± 132.420	6.343 ± 0.712	−1.523 ± 1.817
II	Sodium selenite	3	24.743 ± 3.065	35.182 ± 1.843	0.226 ± 0.045	5.481 ± 0.305	64.627 ± 4.682	96.863 ± 17.769	235.810 ± 152.991	4.085 ± 0.577	1299.767 ± 86.352	6.579 ± 1.180	−0.029 ± 2.251
III	Yeast selenium	0	23.013 ± 1.750	35.460 ± 2.208	0.186 ± 0.026	6.338 ± 1.395	69.760 ± 2.606	123.900 ± 12.551	247.447 ± 128.900	3.685 ± 0.465	1522.067 ± 117.397	7.527 ± 0.592	1.883 ± 0.784
IV	Yeast selenium	3	27.003 ± 0.352	37.082 ± 2.930	0.185 ± 0.015	5.294 ± 0.790	69.215 ± 1.774	116.523 ± 24.647	183.235 ± 64.109	4.345 ± 0.735	1309.367 ± 106.582	5.748 ± 0.433	−0.331 ± 0.673
Main effect	Sodium selenite		23.417 ± 2.478	36.432 ± 2.520	0.211 ± 0.048	5.069 ± 0.616	67.678 ± 4.975	95.297 ± 22.891	193.465 ± 108.719	4.272 ± 0.431	1264.150 ± 107.326	6.461 ± 0.881	−0.776 ± 2.004
	Yeast selenium		25.008 ± 2.460	36.271 ± 2.485	0.185 ± 0.019	5.816 ± 1.164	69.487 ± 2.016	120.212 ± 17.953	215.341 ± 97.607	4.015 ± 0.658	1415.717 ± 153.717	6.638 ± 1.079	0.776 ± 1.378
		0	22.552 ± 1.323	36.572 ± 2.559	0.191 ± 0.038	5.497 ± 1.328	70.245 ± 2.793	108.815 ± 27.034	199.283 ± 98.668	4.072 ± 0.526	1375.300 ± 195.896	6.935 ± 0.874	0.180 ± 2.246
		3	25.873 ± 2.311	36.132 ± 2.424	0.206 ± 0.038	5.388 ± 0.545	66.921 ± 4.043	106.693 ± 22.028	209.523 ± 108.792	4.215 ± 0.608	1304.567 ± 86.915	6.163 ± 0.916	−0.180 ± 1.495
*p*-value	Selenium source		0.168	0.913	0.285	0.174	0.372	0.094	0.73	0.422	0.047	0.705	0.118
	Probiotic preparation		0.013	0.767	0.52	0.833	0.12	0.876	0.871	0.652	0.306	0.125	0.695
	Selenium source + probiotic preparation		0.543	0.188	0.489	0.099	0.184	0.699	0.258	0.129	0.059	0.056	0.07

In contrast, serum antioxidant parameters were more strongly influenced by dietary treatments. As shown in Table 5, selenium source significantly affected T-AOC (*p* = 0.043), MDA (*p* = 0.005), and GSH (*p* < 0.001). Micro-ecological probiotic supplementation significantly affected SOD (*p* = 0.032) and GSH (*p* < 0.001), while the interaction between selenium source and micro-ecological probiotic supplementation had a highly significant effect on GSH (*p* = 0.002). Furthermore, selenium source, micro-ecological probiotic supplementation, and their interaction all significantly affected the PC1 value of antioxidant traits (*p* < 0.001, *p* = 0.003, and *p* < 0.001, respectively). These results indicate that antioxidant capacity was highly responsive to both selenium source and micro-ecological probiotic supplementation, and they were consistent with the clear treatment-related clustering observed in the PCA plot of antioxidant parameters.

**Table 5:** Effects of selenium source and probiotic supplementation on serum antioxidant parameters of beef cattle.

Treatment group	Selenium source	Probiotic preparation/%	T-AOC (mM)	MDA (nmol/mL)	CAT (U/mL)	SOD (U/ml)	GSH (U/mL)	Antioxidant_PC1
I	Sodium selenite	0	0.167 ± 0.044	2.491 ± 0.279	1.602 ± 0.806	171.577 ± 10.706	163.806 ± 18.496	−2.417 ± 0.569
II	Sodium selenite	3	0.233 ± 0.028	2.346 ± 0.354	2.902 ± 0.698	193.792 ± 20.368	285.806 ± 16.461	0.127 ± 0.110
III	Yeast selenium	0	0.283 ± 0.072	1.423 ± 0.277	3.635 ± 0.349	173.940 ± 12.867	282.581 ± 27.097	1.275 ± 0.654
IV	Yeast selenium	3	0.250 ± 0.036	1.859 ± 0.454	3.522 ± 1.985	191.901 ± 4.558	303.871 ± 13.409	1.015 ± 0.376
Main effect	Sodium selenite		0.200 ± 0.049	2.418 ± 0.296	2.252 ± 0.981	182.684 ± 18.970	224.806 ± 68.633	−1.145 ± 1.441
	Yeast selenium		0.267 ± 0.054	1.641 ± 0.412	3.578 ± 1.276	182.921 ± 13.089	293.226 ± 22.396	1.145 ± 0.498
		0	0.225 ± 0.083	1.957 ± 0.635	2.618 ± 1.244	172.758 ± 10.665	223.194 ± 68.284	−0.571 ± 2.095
		3	0.242 ± 0.030	2.102 ± 0.452	3.212 ± 1.373	192.847 ± 13.241	294.839 ± 16.680	0.571 ± 0.546
*p*-value	Selenium source		0.043	0.005	0.079	0.976	<0.001	<0.001
	Probiotic Preparation		0.572	0.492	0.394	0.032	<0.001	0.003
	Selenium source + probiotic preparation		0.113	0.187	0.314	0.79	0.002	<0.001

### 3.3 Effects of selenium source and micro-ecological probiotic supplementation on rumen microbial abundance

**Figure 3:**
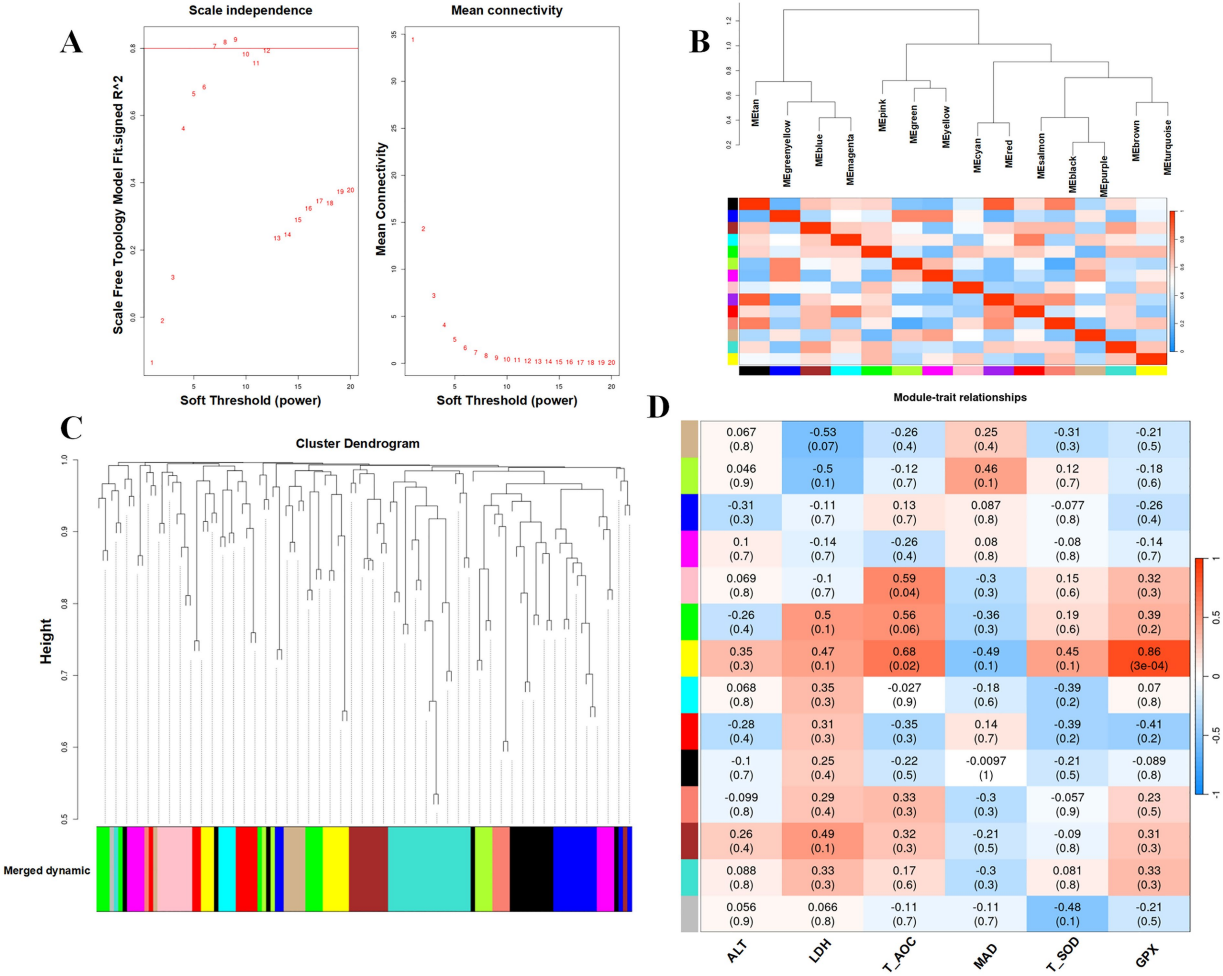
WGCNA analysis of genus-level rumen microbiota and serum traits. **(A)** Selection of soft-thresholding power for network construction. **(B,C)** Identification of microbial co-abundance modules based on hierarchical clustering and topological overlap. **(D)** Correlations between microbial module eigengenes and serum biochemical or antioxidant indices.

To investigate whether rumen microbiota may contribute to treatment-induced phenotypic changes, rumen microbial communities were analyzed in the selected samples. After taxonomic classification and genus-level aggregation, 123 genera were retained for downstream analysis. Relative abundance data were subjected to CLR transformation and used for WGCNA. As shown in Figure 3A, the soft-thresholding power of 7 was selected as the optimal power for network construction. Under this threshold, 14 microbial co-abundance modules were identified, and these modules could be clearly distinguished from each other based on hierarchical clustering and topological overlap patterns (Figures 3B,C). Because rumen microorganisms may indirectly influence growth performance by regulating serum biochemical and antioxidant status, the serum indices significantly affected by selenium source or micro-ecological probiotic supplementation, as identified in Tables 4, 5, were further correlated with microbial module eigengenes. The module–trait relationship analysis showed that T-AOC was significantly associated with the MEpink (*p* = 0.04) and MEyellow modules (*p* = 0.02), while GSH was highly significantly associated with the MEyellow module (*p* = 0.0003). These results suggest that specific microbial modules may be closely linked to host antioxidant regulation. The MEpink module contained eight genera, including *Eubacterium ventriosum* group, Aeriscardovia, Aminipila, Colidextribacter, Schwartzia, SP3-e08, Synergistes, and Treponema. The MEyellow module contained nine genera, including *Eubacterium sulci* group, Acutalibacter, Defluviitaleaceae UCG-011, Desulfovibrio, Paeniclostridium, Papillibacter, Romboutsia, Turicibacter, and UCG-002. These genera may represent key microbial taxa associated with serum antioxidant parameters.

### 3.4 Identification of key microbial taxa associated with antioxidant indices

To further identify candidate microorganisms that may respond to dietary treatments and contribute to host antioxidant status, the 17 genera within the MEpink and MEyellow modules were subjected to two-way ANOVA to evaluate the main effects of selenium source and micro-ecological probiotic supplementation, as well as their interaction. As shown in Table 6, five genera were significantly affected by at least one main effect or interaction effect, including *E. sulci* group, Paeniclostridium, Papillibacter, Romboutsia, and Turicibacter. Specifically, selenium source significantly affected the abundance of *E. sulci* group (*p* = 0.005), Paeniclostridium (*p* = 0.024), Papillibacter (*p* = 0.004), and Romboutsia (*p* = 0.019). Probiotic supplementation significantly affected Paeniclostridium (*p* = 0.001) and Turicibacter (*p* = 0.026). In addition, significant selenium source × probiotic supplementation interaction effects were observed for Paeniclostridium (*p* = 0.015) and Turicibacter (*p* = 0.032). These findings indicate that the abundance of these genera was altered by selenium source and/or micro-ecological probiotic supplementation. Combined with the WGCNA results, these treatment-responsive genera may contribute to changes in serum T-AOC and GSH concentrations.

**Table 6:** Effects of selenium source and probiotic supplementation on microbial genera within the MEpink and MEyellow modules.

Selenium source	Probiotic preparation/%	Eubacterium_sulci_group	Eubacterium_ventriosum__group	Acutalibacter	Aeriscardovia	Aminipila	Colidextribacter	Defluviitaleaceae UCG-011	Desulfovibrio	Paeniclostridium	Papillibacter	Romboutsia	Schwartzia	SP3-e08	Synergistes	Treponema	Turicibacter	UCG-002
Sodium selenite	0	0.466 ± 0.26	2.361 ± 1.101	−2.277 ± 1.041	−2.322 ± 0.967	−2.277 ± 1.041	−1.389 ± 1.267	−0.325 ± 0.58	1.647 ± 0.253	0.556 ± 0.684	−0.987 ± 0.431	0.636 ± 1.102	−1.398 ± 1.121	−2.277 ± 1.041	−2.508 ± 0.663	1.288 ± 0.441	−0.034 ± 0.96	0.231 ± 2.521
Sodium selenite	3	0.689 ± 0.27	2.179 ± 0.049	−0.605 ± 0.884	−2.174 ± 1.26	−2.27 ± 1.096	−2.021 ± 1.522	0.206 ± 0.972	1.127 ± 0.523	−3.002 ± 0.27	−0.628 ± 0.469	−0.713 ± 0.348	−0.832 ± 1.274	−1.817 ± 1.178	−3.002 ± 0.27	0.768 ± 0.932	−2.636 ± 0.484	2.979 ± 1.492
Yeast selenium	0	1.076 ± 0.311	2.223 ± 0.679	−0.887 ± 0.451	−2.049 ± 1.188	−2.116 ± 1.072	−0.961 ± 1.572	0.807 ± 0.632	0.934 ± 0.28	−2.02 ± 1.238	0.123 ± 0.669	−2.2 ± 0.927	−1.005 ± 1.801	−1.603 ± 1.046	−2.312 ± 0.733	1.471 ± 0.529	−2.2 ± 0.927	3.179 ± 0.553
Yeast selenium	3	1.302 ± 0.265	1.951 ± 0.385	−1.274 ± 0.925	−1.17 ± 0.297	−0.962 ± 1.213	−0.078 ± 1.952	0.296 ± 0.677	1.259 ± 0.383	−2.862 ± 0.473	0.461 ± 0.15	−1.476 ± 1.51	−1.917 ± 2.109	−0.9 ± 1.448	−2.213 ± 1	0.64 ± 0.267	−2.265 ± 0.92	2.284 ± 1.142
Sodium selenite		0.578 ± 0.267	2.27 ± 0.704	−1.441 ± 1.259	−2.248 ± 1.007	−2.274 ± 0.956	−1.705 ± 1.3	−0.06 ± 0.772	1.387 ± 0.465	−1.223 ± 2.004	−0.807 ± 0.449	−0.039 ± 1.04	−1.115 ± 1.117	−2.047 ± 1.026	−2.755 ± 0.527	1.028 ± 0.711	−1.335 ± 1.579	1.605 ± 2.387
Yeast selenium		1.189 ± 0.287	2.087 ± 0.516	−1.08 ± 0.685	−1.61 ± 0.912	−1.539 ± 1.203	−0.519 ± 1.657	0.552 ± 0.649	1.097 ± 0.349	−2.441 ± 0.957	0.292 ± 0.472	−1.838 ± 1.189	−1.461 ± 1.824	−1.251 ± 1.193	−2.263 ± 0.786	1.055 ± 0.589	−2.232 ± 0.827	2.731 ± 0.941
	0	0.771 ± 0.421	2.292 ± 0.822	−1.582 ± 1.046	−2.186 ± 0.98	−2.197 ± 0.949	−1.175 ± 1.298	0.241 ± 0.824	1.291 ± 0.458	−0.732 ± 1.671	−0.432 ± 0.789	−0.782 ± 1.801	−1.202 ± 1.359	−1.94 ± 1.004	−2.41 ± 0.634	1.379 ± 0.447	−1.117 ± 1.456	1.705 ± 2.296
	3	0.996 ± 0.413	2.065 ± 0.275	−0.94 ± 0.889	−1.672 ± 0.986	−1.616 ± 1.258	−1.049 ± 1.893	0.251 ± 0.751	1.193 ± 0.416	−2.932 ± 0.353	−0.083 ± 0.673	−1.094 ± 1.065	−1.375 ± 1.668	−1.359 ± 1.283	−2.608 ± 0.785	0.704 ± 0.617	−2.45 ± 0.688	2.632 ± 1.248
Selenium source		0.005096361	0.651806375	0.486108003	0.302191803	0.28360385	0.234551243	0.185605676	0.216368795	0.023767697	0.003601297	0.018543331	0.721598068	0.279830772	0.267278252	0.938345386	0.10348466	0.256417672
Probiotic preparation		0.198631067	0.576431165	0.229506729	0.400879227	0.390026944	0.895053895	0.982145321	0.665034793	0.001015163	0.233254569	0.622560946	0.858086749	0.421773085	0.645411809	0.084868468	0.025822881	0.344086791
Selenium source + probiotic preparation		0.989157573	0.91129225	0.070403258	0.545110414	0.395779598	0.435121688	0.252497599	0.086784838	0.014543023	0.968920677	0.127942776	0.453942047	0.864065515	0.493540574	0.663078938	0.031705301	0.083497575

Because WGCNA evaluates associations at the module level, we next focused on the five key genera that were located in the significant modules and were also affected by dietary treatments. Correlation analysis was performed between these genera and the antioxidant indices T-AOC and GSH. As shown in Table 7, Romboutsia was significantly correlated with T-AOC (*p* = 0.02), whereas the other four genera showed significant correlations with GSH. Specifically, *E. sulci* group and Papillibacter were positively correlated with GSH (*p* = 0.002, *p* = 0.0064, respectively), whereas Paeniclostridium and Turicibacter were negatively correlated with GSH (p = 0.02, *p* = 0.04, respectively).

**Table 7:** Correlations between key candidate genera and serum antioxidant indices T-AOC and GSH.

Microbe	T_AOC_cor^1^	T_AOC_cor_p^2^	GSH_cor	GSH_cor_p
Eubacterium	0.52	0.09	0.8	0.002
Sulci group				
Paeniclostridium	−0.39	0.21	−0.65	0.02
Papillibacter	0.43	0.17	0.74	0.0064
Romboutsia	−0.67	0.02	−0.48	0.11
Turicibacter	−0.26	0.42	−0.6	0.04

^1^cor, correlation coefficient; ^2^p, *p*-value of the correlation coefficient.

**Figure 4:**
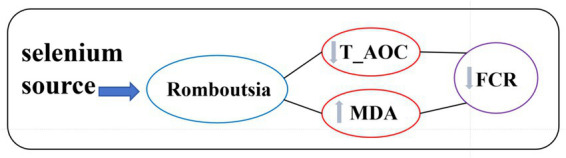
Proposed mechanism by which selenium source affects FCR through rumen microbiota and antioxidant status.

Among these candidate genera, Romboutsia was of particular interest. T-AOC was significantly associated with FCR in the regression analysis (Figure 1C), and T-AOC was also significantly affected by selenium source (*p* = 0.043; Table 5). Meanwhile, Romboutsia abundance was significantly affected by selenium source (*p* = 0.019; Table 6) and was significantly correlated with T-AOC (r = −0.67, *p* = 0.020; Table 7). In addition, Romboutsia was significantly correlated with MDA at the individual genus level (*r* = 0.569, *p* = 0.044). Since both T-AOC and MDA were associated with FCR and were affected by selenium source, these results suggest that Romboutsia may serve as a microbial link between selenium source, antioxidant status, and feed efficiency. Taken together, these findings support a potential mechanistic pathway in which selenium source alters the abundance of Romboutsia, which may subsequently influence serum antioxidant parameters, particularly T-AOC and MDA, ultimately contributing to differences in FCR among treatment groups (Figure 4).

## 4 Discussion

### 4.1 Effects of selenium source and micro-ecological probiotics on growth performance of beef cattle

In commercial beef cattle production, enhancing economic returns largely depends on improving key performance metrics, including ADG, feed efficiency, and final body weight. Numerous studies have demonstrated that selenium, particularly in its organic form (yeast selenium), and micro-ecological micro-ecological preparations can improve the production performance of ruminants. Shin et al. (21) reported that supplementing selenium can significantly increase weight gain in cattle, and Grossi et al. (22) further found that organic selenium elevates ADG in beef cattle. This is in line with the idea that selenium supports metabolic functions essential for optimal growth, including antioxidant activity and immune function. In our study, selenium source had a significant effect on FCR, with sodium selenite supplementation leading to a significant reduction in FCR compared to yeast selenium. The observed reduction in FCR in the sodium selenite groups would not be interpreted as evidence that sodium selenite has greater bioavailability than yeast selenium. In general, yeast selenium, as an organic selenium source, has higher bioavailability and tissue retention than inorganic selenium because selenomethionine can be incorporated into body proteins in place of methionine and serve as a selenium reserve (23). In contrast, sodium selenite is absorbed relatively rapidly but is readily reduced to selenide and either used for immediate selenoprotein synthesis or excreted, resulting in limited long-term tissue storage compared with organic selenium (24). Therefore, the lower FCR observed in the sodium selenite groups may represent a physiological response under the current 37-day feeding trial conditions, potentially involving short-term changes in antioxidant status, energy metabolism, and rumen microbial activity, rather than reflecting superior selenium bioavailability. Longer-term studies with larger sample sizes and measurements of tissue selenium deposition are needed to further validate the underlying mechanisms by which different selenium sources influence feed efficiency.

It is also important to consider the interaction between selenium source and micro-ecological probiotics (13). While micro-ecological probiotics such as Lactobacillus and *S. cerevisiae* enhance rumen fermentation, microbial balance, and nutrient absorption, the combined effect with yeast selenium was not as pronounced in improving feed efficiency as with sodium selenite. This could be because micro-ecological probiotics may have a greater synergistic effect with inorganic selenium, which acts more rapidly in enhancing metabolic functions. Thus, the combination of micro-ecological probiotics with sodium selenite may offer a more effective strategy for improving feed efficiency in beef cattle (25).

### 4.2 Effects of selenium source and micro-ecological probiotics on serum biochemical parameters in beef cattle

Serum biochemical indices are critical indicators for assessing animal health and metabolic status (26). Previous studies have shown that micro-ecological probiotics can modulate serum GLU, TC, and TG levels by enhancing digestive enzyme activity and improving carbohydrate absorption. For example, Yasmin et al. (27) found that compound micro-ecological preparations significantly increased serum GLU, TC, and TG in finishing cattle, likely due to lactic acid bacteria enhancing amylase activity. In contrast, our study found that selenium source had a significant effect on serum liver enzymes, particularly ALT and LDH. ALT is an important enzyme involved in protein metabolism, reflecting both protein catabolism and anabolism, as well as the liver’s functional status (28). Elevated ALT levels are often associated with liver stress or damage, but values within the normal range indicate healthy liver function. Zaitsev et al. (29) reported that normal serum ALT activity in beef cattle falls within 7–32 *μ*/L, and the ALT levels observed in our study were within this range for all groups. However, sodium selenite supplementation significantly reduced ALT levels compared to yeast selenium, suggesting that inorganic selenium may alleviate metabolic stress in the liver more effectively than organic selenium.

LDH, another critical enzyme involved in carbohydrate metabolism, also exhibited significant reductions with sodium selenite supplementation. LDH catalyzes the conversion of lactate to pyruvate, which is crucial for energy production during amino acid and lipid metabolism (30). The reduced levels of LDH with sodium selenite may indicate improved energy metabolism efficiency and less oxidative stress during metabolism, contributing to overall metabolic health. These findings suggest that inorganic selenium may provide more immediate metabolic benefits, leading to better liver and muscle function compared to organic selenium.

### 4.3 Effects of selenium source and micro-ecological probiotics on antioxidant capacity in beef cattle

Under normal physiological conditions, beef cattle possess a robust antioxidant system to protect against oxidative stress. This system includes enzymatic components such as GSH, CAT, and SOD, all of which are essential for neutralizing reactive oxygen species (ROS) (31). Numerous studies have confirmed that selenium supplementation enhances antioxidant capacity by increasing the activity of GSH, a key selenoprotein involved in oxidative defense. Gunter et al. (32) demonstrated that selenium supplementation significantly increased plasma GSH concentrations, with yeast selenium showing the most pronounced effects. Our study confirmed that selenium source significantly influenced several antioxidant indices, including T-AOC, MDA, SOD, and GSH. Both selenium source and micro-ecological probiotic supplementation interacted to modulate these antioxidant parameters, with yeast selenium having a more pronounced effect on GSH levels. Yeast selenium likely improves antioxidant status by enhancing selenoprotein synthesis more efficiently than sodium selenite, which, while more bioavailable, has a less sustained effect (33).

Micro-ecological probiotic supplementation also affected antioxidant indices, especially GSH, highlighting the beneficial role of micro-ecological probiotics in maintaining oxidative balance. Micro-ecological probiotics likely exert their effects by stabilizing the rumen microbiota, reducing the production of harmful metabolites, and promoting the synthesis of beneficial antioxidants (34). The combined effect of selenium and micro-ecological probiotics may thus offer a comprehensive strategy to optimize oxidative defense in beef cattle.

### 4.4 Effects of selenium source and micro-ecological probiotics on microbial abundance

The rumen microbiota plays a pivotal role in nutrient digestion, immune modulation, and metabolic regulation. In our study, WGCNA revealed that selenium source and micro-ecological probiotic supplementation significantly affected the microbial composition, with distinct microbial modules associated with antioxidant indices. Specifically, the MEpink and MEyellow modules, which were significantly correlated with T-AOC and GSH, contained genera such as Romboutsia, Paeniclostridium, and Turicibacter.

These microbial genera are involved in key metabolic processes such as fiber degradation, short-chain fatty acid production, and amino acid metabolism (35). Their altered abundance in response to selenium source and micro-ecological probiotics suggests that microbial metabolism may influence host antioxidant capacity, thereby affecting growth performance. The presence of Romboutsia, for example, was significantly correlated with T-AOC, indicating that this genus might play a role in enhancing antioxidant capacity and improving feed efficiency (36, 37).

### 4.5 Proposed mechanistic pathway linking selenium source, microbiota, and growth performance

Our findings suggest a potential mechanistic pathway in which selenium source modulates the abundance of Romboutsia in the rumen, which subsequently influences serum antioxidant parameters, such as T-AOC and MDA. These antioxidants, in turn, affect FCR. Specifically, we observed that higher T-AOC levels were positively correlated with FCR, whereas MDA levels were negatively correlated with FCR. Interestingly, the positive correlation between T-AOC and FCR and the negative correlation between MDA and FCR observed in this study contrast with some previous reports (38). Previous research has shown that high levels of MDA, a marker of lipid peroxidation, are typically associated with poor feed efficiency (39). This discrepancy with previous findings may be explained by the fact that animals with high feed efficiency (low FCR) are characterized by an exceptionally efficient conversion of nutrients into body mass. Due to more thorough and rapid metabolism, MDA, an end product of lipid peroxidation, accumulates to higher levels in high-efficiency individuals. Meanwhile, in the face of continuously generated high levels of reactive oxygen species (ROS), the body’s antioxidant reserves, such as GSH, vitamins C and E, and antioxidant enzymes, are constantly mobilized and consumed. As a result, the residual, or “standing stock,” T-AOC detected in the circulating blood appears relatively lower. This implies that the biochemical and antioxidant indicators measured in serum may largely represent the quantities remaining after systemic metabolism, underscoring the need for rigorous evaluation of sampling time points and physiological stages. Furthermore, our study suggests that, in this context, selenium supplementation may modulate oxidative stress in a manner that enhances feed efficiency, potentially through the mediation of Romboutsia-driven metabolic pathways.

Taken together, these results provide new insights into the complex relationship between selenium supplementation, microbiota composition, and growth performance in beef cattle. By modulating rumen microbial communities and enhancing antioxidant status, selenium supplementation, particularly in its inorganic form, may represent an effective strategy to improve feed efficiency and overall performance in beef cattle. One limitation of the present study is the relatively small sample size, particularly for the serum biochemical, antioxidant, and rumen microbiota analyses. Although significant treatment effects were observed, future studies with larger sample sizes would be valuable to further validate and strengthen the robustness of the present findings.

## 5 Conclusion

Selenium source and micro-ecological probiotic supplementation jointly affect the antioxidant capacity and rumen microbial structure of beef cattle. Selenium source significantly influences feed conversion ratio, with sodium selenite improving feed efficiency better than yeast selenium. Key microbial genera such as Romboutsia, Paeniclostridium, and Turicibacter are closely associated with host antioxidant indices, and selenium source could possibly regulate growth performance through a microbiota–antioxidant axis, particularly Romboutsia-mediated regulation of oxidative status. These findings provide new insights into nutritional strategies for improving beef cattle production.

## Data Availability

The 16S rRNA raw data supporting the conclusions of this article have been uploaded to the Figshare repository: https://doi.org/10.6084/m9.figshare.32934602.
